# Construction of nursing-sensitive quality indicator system for cardiac rehabilitation of patients undergoing percutaneous coronary intervention based on structure-process-outcome model

**DOI:** 10.1186/s12912-023-01618-w

**Published:** 2023-12-04

**Authors:** Lei Kang, Min-hui Wang, Sheng-jia Wu

**Affiliations:** Cardiovascular Center, Ruijin Hospital, Shanghai Jiao Tong University School of Medicine, Shanghai, 200025 China

**Keywords:** Cardiac rehabilitation (CR) after percutaneous coronary intervention (PCI), Nursing-sensitive, Quality indicator, Structure-process-outcome model, Delphi method

## Abstract

**Background:**

Coronary heart disease (CHD) is a cardiovascular disease with high mortality. At present, percutaneous coronary intervention (PCI) is considered as the main effective treatment for CHD due to less trauma, shorter course of treatment, and better curative effect. However, PCI alone is not a permanent cure, so cardiac rehabilitation (CR) is needed for a supplement. Nowadays, the evaluation of the nursing-sensitive quality of CR after PCI focuses on the outcomes of patients, lacks a complete evaluation indicator system, and is prone to problems such as nursing management imbalance.

**Objective:**

A scientific, sensitive, comprehensive and practical nursing-sensitive quality indicator system based on the structure-process-outcome model was constructed to provide a reference for evaluating nursing-sensitive quality of CR after PCI.

**Methods:**

Firstly, through literature analysis and semi-structured interview, the indicator system was collected, screened and determined. Then, the framework of the indicator system was established, and the draft of nursing-sensitive quality indicator system of CR after PCI was formed. Subsequently, the nursing-sensitive quality indicator system of CR after PCI was initially established using Delphi method. Finally, the specific weight was determined by analytic hierarchy process (AHP), and the nursing-sensitive quality indicator system of CR after PCI was established and perfected.

**Results:**

Two rounds of expert consultations were separately given 15 questionnaires, and all these questionnaires were returned, with a questionnaire response rate of 100%. Such result indicated that experts were highly motivated. Besides, the authoritative coefficients for two rounds of expert consultations were 0.865 and 0.888, and the coordination coefficients were 0.491 and 0.522, respectively. Hence, the experts’ authority and coordination were high and the results were reliable. After the second round of expert consultation, the nursing-sensitive quality indicator system of CR after PCI was established, eventually. This system consisted of 3 first-level indicators (structural indicator, process indicator and outcome indicator), 11 s-level indicators and 29 third-level indicators.

**Conclusion:**

A relatively complete and reliable nursing-sensitive quality indicator system of CR after PCI has been established in this study. Such system is scientific and reliable and can provide a reference for the evaluation of clinical teaching quality of CR after PCI.

**Supplementary Information:**

The online version contains supplementary material available at 10.1186/s12912-023-01618-w.

## Introduction

Coronary heart disease (CHD), also known as coronary artery disease, is a very common cardiovascular disease. Specifically, CHD refers to coronary artery stenosis due to plaque or atherosclerosis in the arterial wall, thereby affecting the blood supply to the heart [1]. At present, CHD is the leading cause of death in the world, and its morbidity and mortality are rising significantly, especially in developing countries [2]. Percutaneous coronary intervention (PCI), also known as coronary angioplasty, is considered as a common interventional procedure used to treat CHD [3]. However, PCI can only temporarily restore blood supply to the heart, but does not fundamentally prevent the formation of atherosclerotic plaques and coronary artery stenosis, that is, the so-called palliative. Studies have reported in-stent restenosis rates of 8.21% [4] and cardiovascular endpoint rates of 14.4% within 1 year after PCI, and 2-year adverse cardiac events as high as 26.5%. Postoperative complications of PCI seriously affects the quality of life of patients [5, 6]. Therefore, health management of patients after PCI is a question worth discussing to prevent the occurrence of adverse cardiovascular events.

Cardiac rehabilitation (CR) is a rehabilitation treatment method designed to improve heart health after heart surgery. As a treatment containing rehabilitation evaluation, exercise therapy, diet guidance and so on, CR can help patients to better restore their heart function, improve their quality of life and reduce complications [7]. In the process of CR in hospital, nursing quality is an important part of hospital management quality. Under the background of big data analysis in hospital management, nursing-sensitive quality indicator can guide nurses to identify nursing problems and take corresponding measures to improve nursing quality [8]. In July 2016, the *Handbook of Nursing-sensitive Quality Indicators (2016 Edition)* compiled by National Institute of Hospital Administration proposed 12 items of nursing-sensitive quality indicators. These indicators have become the focus of hospital nursing quality managers nationwide. [9]. Nursing-sensitive quality indicator is the procedure and outcome of nursing service provided to patients, which is evaluated by nursing data, quantitative analysis, and monitoring of various functional qualities (including nursing management and clinical practice) that affect patients’ outcomes. Donabedian proposed the theoretical model of “three-dimensional quality structure” as early as 1992, that was, medical quality was divided into three dimensions, including structure, process and outcome. This theoretical model has became the main framework for constructing nursing-sensitive quality indicators [10]. At present, the evaluation of rehabilitation nursing quality after PCI mainly focuses on the outcome of patients and lacks a complete evaluation indicator system (structural quality and process quality). Moreover, such evaluation only concentrates on the outcome quality but not pays attention to the structural quality and process quality, so it is prone to problems such as nursing management imbalance [11]. Therefore, this study intends to build a scientific, sensitive, comprehensive and practical nursing sensitivity quality index system based on structure-process-outcome model by using Delphi expert correspondence method on the basis of a large number of literature reviews and semi-structured interviews. The objective of this study was to improve the quality of clinical nursing and provide a basis for future research on the safety and quality management of CR nursing after PCI.

## Methods

### Study design

#### The establishment and duty definition of research team

This research team consisted of 15 members, and the leader was the chief nurse of the CR center after PCI and engaged in the management of CR after PCI for 22 years. The members of this research consisted of 5 directors and 4 head nurses, of which included 4 doctors and 3 masters, and the rest were bachelors. All nurses had more than 7 years of rich front-line work experience and nursing management ability. The members of the research team were mainly responsible for consulting related literature, determining the expert consultation questionnaires, reclaiming, screening and sorting out the feedback from experts, and constructing the nursing-sensitive quality indicator system of CR after PCI through the evaluation and statistical analysis of the outcomes of consultation questionnaires.

### Literature research

Literature was searched form PubMed, China National Knowledge Infrastructure (CNKI), Wanfang database and Web of Science taking “cardiac rehabilitation (CR) after percutaneous coronary intervention (PCI)”, “nursing-sensitive”, “quality indicator”, “structure-process-outcome model”, and “Delphi method” as keywords. The search time was set from the database establishment to April 2023. The inclusion and exclusion criteria of literature was shown as follows [12]. The inclusion criteria included literature (1) involving CR after PCI; (2) related to nursing-sensitive indicators; (3) in Chinese and English; (4) related to clinical practice guidelines and expert consensus; and (5) Johns Hopkins Evidence-Based Practice A and B literature. The exclusion criteria were composed of literature (1) that failed to obtain the original text and contact the author; (2) with incomplete or unusable data. In this paper, 15 studies were searched, sorted, screened, cross-checked and discussed together. Finally, 40 items of indicators were acquired, including 10 structural indicators, 13 process indicators and 17 outcome indicators.

### Semi-structured interviews

The data were collected through semi-structured interviews, and some literature materials were enrolled for a supplement (Supplementary material 1 and Supplementary material 2). During the interview, the researcher flexibly adjusted the order, method and topic of questioning according to specific situations and interview outlines. Besides, the details of valuable questions were asked to deeply understand the opinions of the interviewed experts. The inclusion and exclusion criteria of interviewees were shown as follows [13]. The inclusion criteria included interviewees (1) serving as head nurses more than 3 years; (2) understanding the knowledge of nursing quality evaluation and volunteering to participate in the interview; (3) with clinical nursing backbone related clinical working years ≥ 5 years; (4) with a professional title of chief nurse; (5) with a certain understanding of nursing quality or participating in similar inspections and activities before; (6) who informed consent and were willing to cooperate with the interview. As for exclusion criteria, interviewees who quit the interview halfway were excluded from this study. In addition, the interviews were analyzed and sorted out, and the expert consultation questionnaires for nursing-sensitive quality indicators of CR were compiled according to the theoretical model of “structure-process-outcome”.

### Delphi method

Firstly, experts were screened according to Delphi method [14], mainly including age, working year, working field, education background, professional title, as well as the judgment basis and familiarity degree to indicators. Briefly, the standards of hospitals experts engaged in were Grade III Level A hospitals (The highest level of hospital in China). Concerning post standard of expert work, the head nurse must have 5 years of relevant nursing management experience at least, nurses responsible for CR after PCI must have 10 years of relevant clinical experience at least, and nursing quality management experts must work in the field of quality management in nursing department for more than 5 years. Besides, the standard of professional titles was defined as the title of chief nurse or above. Nursing experts participating in this project must have a education background of bachelor or above. Moreover, experts were required a rigorous working attitude and high enthusiasms for the questions consulted, could ensure to participate in at least two rounds of expert consultations, and were able to answer difficult questions in the process of expert consultations. Subsequently, related questionnaires were distributed and reclaimed. Finally, the indicators were screened. Specifically, based on the Delphi method, the indicators were screened by three criteria: indicator importance score, coefficient of variation (CV) and expert’s literal opinions. The indicators with the importance assignment mean > 4.0 and CV<0.20 were retained [15].

### Statistical method

SPSS 23.0 and Excel software were used for data entry and statistical analysis. The basic data of experts were described by means, standard deviation (SD), name and percentage, and the significance scores of indicators at all levels were expressed by mean ± SD. The positive coefficient was expressed by the questionnaire response rate; the expert authority was expressed by authoritative coefficients; the concentration degree of expert opinions was expressed by the importance assignment mean; the coordination degree of expert opinions was expressed by Kendall harmony coefficient. The analytic hierarchy process (AHP) was used to determine the weight of indicators at all levels, and P < 0.05 indicated a significant difference.

## Results

### General information for experts

As shown in Tables 1 and 15 experts selected for this study came from six provinces and cities across the country and involved the areas of nursing management, clinical nursing, and nursing education. The age of these experts ranged from 33 to 52 years, with a mean age of 41 ± 6.03 years; length of service ranged from 6 to 22 years, with a mean length of 13.47 ± 4.73 years. All experts had professional title of secondary senior positions or above, including 5 professors or chief nurses (33.3%) and 10 associate professors or deputy chief nurses (66.7%).

**Table 1:** General information for experts

Variables	Frequency (%)
Professional title	
Nursing management	4 (26.67)
Clinical nursing	7 (46.67)
Nursing education	4 (26.67)
Provinces and cities	
Shanghai	6 (40.00)
Zhejiang	3 (20.00)
Jiangsu	3 (20.00)
Beijing	1 (6.67)
Shandong	1 (6.67)
Guangdong	1 (6.67)
Age	41.00 ± 6.03
Length of service	13.47 ± 4.73
Professional titles	
Professors or chief nurses	5 (33.3)
Associate professors or deputy chief nurses	10 (66.7)

### Enthusiasm, authority and coordination of experts

As shown in Table 2, two rounds of expert consultations were conducted in this study; 15 questionnaires were separately distributed to two rounds of expert consultations, all questionnaires were reclaimed, and the questionnaire response rate was 100%. Such result indicated that experts were highly motivated. Additionally, Kendall harmony coefficients of the two rounds of experts were 0.491 and 0.522, respectively, and the P values were both less than 0.001; the judgment basses were 0.863 and 0.897; the familiarity degrees were 0.867 and 0.888; and the authoritative coefficients were 0.865 and 0.888 (Table 3). The above outcomes suggested high degree of coordination and authority of the opinions of the experts consulted and reliability of the results of the consultations.

**Table 2:** Test table for Kendall harmony coefficients

	Overall indicators		
	Kendall	*χ2*	*P*
The first round	0.491	250.609	0.000
The second round	0.522	266.081	0.000

**Table 3:** Table of expert review

	Judgment basis	Familiarity degree	Authoritative coefficient
The first round	0.863	0.867	0.865
The second round	0.897	0.888	0.888

### Results of expert consultations

As displayed in Table 4, after the first round of expert consultation, no indicator was modified, adjusted and added, excepted 6 indicators deleted. After the second round of expert consultation, 6 indicators were deleted, and the nursing-sensitive quality indicator system of CR after PCI was eventually established, including 3 first-level indicators, 11 s-level indicators and 29 third-level indicators. By further observing Table 4, we can find that in terms of structural indicators, the hospital has a high level of resources and equipment, professional staffing, and standardized management of the hospital system are conducive to the rehabilitation of patients. In addition, in the nursing process, the personalized nursing plan and implementation, and the nursing staff’s deep care for patients are all sensitive quality indicators of cardiac rehabilitation nursing after PCI. In terms of outcome indicators, patients’ rehabilitation, prognosis, medical burden and satisfaction were found to be important indicators to evaluate the sensitivity quality of cardiac rehabilitation nursing after PCI. The AHP was employed to calculate the weight of each indicator, and the consistency ratio of all indicators at all levels was less than 0.1, so the setting of indicator weight was reasonable.

**Table 4:** Nursing-sensitive quality indicators for cardiac rehabilitation after percutaneous coronary intervention

First-level indicator	Second-level indicator	Third-level indicator	Weight	Combined weight
Istructural indicator (0.375)	I-1 resource equipment (0.320)	I-1-1 equipment integrity	0.394	0.047
		I-1-2 advancement in equipment	0.344	0.041
	I-2 manning (0.354)	I-2-1 proportion of cardiac nurses	0.324	0.043
		I-2-2 proportion of professional titles of nurses	0.324	0.043
		I-2-3 professional skill level and experience of nurses	0.343	0.046
	I-3 institutional norms (0.330)	I-3-1 nursing management system	0.382	0.047
		I-3-2 nursing process norm	0.361	0.045
II process indicator (0.435)	II-1 Nursing plan (0.243)	II-1-1 individualized formulation of nursing plan	1.000	0.106
	II-2 nursing implementation (0.243)	II-2-1 vital signs of patients observed as planned 24 h after surgery	0.340	0.036
		II-2-2 standardization of patients’ postoperative diet plans and oral medications	0.322	0.034
		II-2-3 timely guidance to patients on postoperative self-rehabilitation exercises	0.336	0.036
	II-3 nursing assessment (0.260)	II-3-1 pain score	0.330	0.037
		II-3-2 input of vital signs	0.335	0.038
		II-3-3 record of changes in disease	0.335	0.038
	II-4 nursing communication (0.253)	II-4-1 communication between nurses and patients	0.375	0.041
		II-4-2 communication between nurses and patients’ family members	0.375	0.041
III outcome indicator (0.284)	III-1 rehabilitation situation (0.254)	III-1-1 recovery of daily living ability	0.192	0.014
		III-1-2 pain situation	0.195	0.014
		III-1-3 recovery of cardiopulmonary function	0.201	0.014
		III-1-4 mental state	0.206	0.015
		III-1-5 medication adherence	0.206	0.015
	III-2 prognosis (0.262)	III-2-1 incidence of complications	0.255	0.019
		III-2-2 incidence of recurrent cardiovascular events	0.245	0.018
		III-2-3 re-hospitalization rate	0.2517	0.019
		III-2-4 mortality	0.248	0.018
	III-3 medical burden (0.254)	III-3-1 length of hospitalization	0.378	0.027
		III-3-2 hospitalization expense	0.368	0.027
	III-4 satisfaction (0.229)	III-4-1 patient satisfaction	0.378	0.025
		III-4-2 physician satisfaction	0.368	0.024

## Discussion

Through two rounds of expert consultations, the nursing-sensitive quality indicators of CR after PCI were formed in this study finally. These indicators were composed of 3 structural indicators (resource equipment, manning and institutional norms), 4 process indicators (nursing plan, nursing implementation, nursing assessment and nursing communication) and 4 outcome indicators (rehabilitation situation, prognosis, medical burden and satisfaction).

Structural indicators mainly reveal the influence of stable medical support environment on nursing, including human resource allocation, organizational structure and economic policy [16]. The structural indicators finally determined in this study included 3 s-level indicators and 7 third-level indicators. These 3 s-level indicators consisted of resource equipment, manning and institutional norms. As for 7 third-level indicators, they included equipment integrity, advancement in equipment, the proportion of cardiac nurses, the proportion of professional titles of nurses, the professional skill level and experience of nurses, nursing management system, and nursing process norm. Of them, the weight of equipment integrity (0.394) and the combined weight of equipment integrity and nursing management system (0.047) were the highest. Such outcome indicated that advanced and complete medical equipment is the primary condition for successful implementation of CR after PCI [17]. The next highest weights were management system (0.382) and nursing process norm (0.361). In the management of CR, formulation of a standardized management system, such as establishing a CR team and making rehabilitation goals and plans, is very important. In addition, the nursing process can provide standardized, safe and scientific nursing measures for patients, so it also plays a vital role in the management of CR [18].

Process indicators, referring to patients’ experience or the specific process implemented by nurses, can reflect the specific activities needed in the provision of medical services [19]. Among the 9 third-level process indicators in this study, the highest weight and combination weight were the individualized formulation of nursing plan. Individualized formulation of nursing plan refers to customizing nursing plan suitable for patients according to their specific conditions and needs. Such plan can better meet the physical, psychological and social needs of patients and provide more effective nursing and rehabilitation services. Especially for CR after PCI, individualized nursing plan can be formulated according to postoperative status, cardiac health and personal goals of patients [20]. Moreover, There is a study that individualized nursing plan induces fewer complications and alleviates poor prognosis [21]. Therefore, patients undergoing CR after PCI needs to be provided with unified clinical management and individualized nursing plans, so that the rehabilitation nursing quality and prognosis of patients can be improved.

Outcome indicators mainly include patient satisfaction and overall quality assessment [22]. The outcome indicators determined in this study mainly consisted rehabilitation situation, prognosis, medical burden and satisfaction. Of them, length of hospitalization, hospitalization expense and patient/doctor satisfaction were the third-level outcome indicators with high weight and combined weight. The main reason may be that the ultimate goal of nursing behavior is to shorten length of hospitalization, reduce hospitalization expenses and improve the satisfaction of patients and doctors [23]. In addition, the outcome indicators with higher weights and combined weights were directly related to the process indicators. For instance, individualized formulation of nursing plan is directly correlated with rehabilitation situation, prognosis, medical burden and satisfaction [24]. Therefore, the quality of CR can be effectively improved through monitoring the rehabilitation situation and prognosis, reducing medical burden and improving satisfaction.

This study has provided a more reliable and comprehensive basis for evaluating the quality and safety of CR after PCI. However, there are some demerits in this study. For example, due to the limitation of time and space, we only selected experts from six provinces and cities in China for consultation, and the number of experts selected for consultation was also small. Hence, a stratified sampling will be perform on experts nationwide in future research to improve the representativeness of experts as far as possible.

## Conclusion

To sum up, 29 nursing-sensitive quality indicators of CR after PCI have been successfully constructed in this study based on literature analysis through semi-structured interviews, Delphi method and three-dimensional quality structure model. Due to scientific, reliable and practical characteristics in clinical application, these indicators can be used as the assessment tools for the prognosis of patients and CR after PCI.

### Electronic supplementary material

Below is the link to the electronic supplementary material.

Supplementary Material 1

Supplementary Material 2

## Data Availability

The data that support the findings of this study are available from the corresponding author upon reasonable request.

## Abbreviations

CHD: Coronary heart disease
PCI: Percutaneous coronary intervention
CR: Cardiac rehabilitation
AHP: Analytic hierarchy process
CNKI: China National Knowledge Infrastructure
CV: Coefficient of variation
SD: Standard deviation
