# Supplementary material for: Construction of nursing-sensitive quality indicator system for cardiac rehabilitation of patients undergoing percutaneous coronary intervention based on structure-process-outcome model
Source: BMC Nurs. 2023 Dec 4;22:457. doi: 10.1186/s12912-023-01618-w (PMC10696800; doi:10.1186/s12912-023-01618-w)
Supplement: Supplementary file 1 — Supplementary Material 1 [file 12912_2023_1618_MOESM1_ESM.docx]

**Construction of nursing-sensitive quality indicator system for cardiac rehabilitation of patients undergoing percutaneous coronary intervention based on structure-process-outcome modelⅠ**

**Expert letter inquiry form**

Dear experts!

The purpose of this study is to establish a sensitivity quality index system of cardiac rehabilitation care after PCI based on a structure-process-outcome model to provide a scientific basis for the quality evaluation of cardiac rehabilitation care after PCI. Please help us to complete this expert consultation according to your own knowledge structure and work experience. At the same time, thank you very much for taking time out of your busy schedule to support our work. After the two rounds of consultation, we will give you a certain consultation fee.

**Fill in the form:**

1 The questionnaire is divided into two parts. The first part is the expert basic information table, and the second part is the index importance scoring table. In addition to the scoring, the experts can also put forward supplementary opinions. If you have other indicators to be included, you can fill in the column of "Please supplement" under the indicators.

2 Importance: As you think, the scores of the following indicators are divided into five grades, from low to high, 1,2,3,4 and 5 points respectively, and the scores are all integer.

3 familiarity: You score the familiarity of each indicator in the following table. The score is divided into five levels, from low to high, 1,2,3,4,5 points, and the scores are all integer.

4 Contact person: Wu Shengjia mobile phone: 13564047742; email: wsj 21704 @ rjh.com. Cn; WeChat ID with the mobile phone number. Please ask the experts to give feedback, can send to wechat or email

**Table 1. Basic information of consulting experts**

Name of the medical institution: Shanghai Sixth People's Hospital

Medical institution level: Grade 3 A

1. Gender: (1) male (2) female ☑
2. Date of birth: 75 year 12 moon
3. Degree: (1) doctor; (2) master; (3) bachelor ☑; (4) no degree;

4. Current title: (1) senior; (2) deputy high ☑; (3) intermediate; (4) junior

5. Years of work: (1) <20 years; (2) ☑ for 20-30 years; (3)> =30 years
6. Current positions: (1) director of department; (2) deputy director of department; (3) university teacher; (4) other ☑——

7. Your familiarity with the evaluation of senior health professional titles:
(1) very familiar; (2) familiar; ☑ (3) general; (4) not familiar; (5) very unfamiliar

8. Whether he is a graduate supervisor: (1) doctoral supervisor; (2) master supervisor; (3) no ☑

9. Your judgment basis: influence size on you: a influence degree is large ☑: b influence degree is general; C influence degree is small

| theoretical analysis | Practical work experience | Domestic and foreign counterparts to understand | Intuitive feeling |
| --- | --- | --- | --- |
| A | A | A | B |

**Table 2 Index of importance and familiarity scores**

| **metric** | **Importance level (0-5 points)** | **Familiarity (0-5 points)** | **Your revised opinion** |
| --- | --- | --- | --- |
| **I structure index** |  |  |  |
| I-1 resource equipment |  |  |  |
| I-1-1 equipment completeness | **5** | **5** |  |
| I-1-2 advanced equipment | **5** | **5** |  |
| I-1-3 drug equipment | **5** | **5** |  |
| I-2 Staffing |  |  |  |
| I-2-1 for the proportion of specialist cardiac nurses | **5** | **5** |  |
| I-2-2 The professional title proportion of nursing staff | **5** | **4** |  |
| I-2-3 Professional skill level and experience of the caregivers | **5** | **5** |  |
| I-3 institutional specification |  |  |  |
| I-3-1 Nursing management system | **5** | **5** |  |
| I-3-2 nursing process specification | **5** | **5** |  |
| I-3-3 Compliance with the Code of Care | **5** | **5** |  |
| Rational application of I-3-4 drugs | **5** | **5** |  |
| I-3-5 Safe use of medical devices | **5** | **5** |  |
| **Admidia process index** |  |  |  |
| -1 Care plan | **5** | **5** |  |
| -1-1 Personalized nursing plan formulation | **5** | **5** |  |
| -2 Nursing implementation |  |  |  |
| -2-1 Observe the patients with scheduled vital signs 24 hours after surgery | **5** | **5** |  |
| -2-2 Conduct the postoperative diet plan and oral medication | **5** | **5** |  |
| -2-3 Guide patients to conduct timely postoperative self-rehabilitation exercise | **5** | **5** |  |
| -3 Nursing assessment |  |  |  |
| -3-1 The pain score | **5** | **5** |  |
| -3-2 Entry of vital signs | **5** | **5** |  |
| -3-3 Changes of the condition were recorded | **5** | **5** |  |
| -4 Nursing communication |  |  |  |
| -4-1 Nursing staff to communicate with patients | **5** | **5** |  |
| -4-2 Nursing staff should communicate with their family members | **5** | **5** |  |
| **Outcome indicators:** |  |  |  |
| -1 Rehabilitation status |  |  |  |
| -1-1 Recovery of daily living capacity | **5** | **5** |  |
| -1-2 Pain condition | **5** | **5** |  |
| -1-3 Recovery of cardiopulmonary function | **5** | **5** |  |
| -1-4 Mental state | **5** | **5** |  |
| -1-5 Medication adherence | **5** | **5** |  |
| -1-6 Recovery of physical function | **5** | **5** |  |
| -1-7 Respiratory recovery status | **4** | **4** |  |
| -2 The prognosis situation |  |  |  |
| -2-1 The complication rate | **5** | **5** |  |
| -2-2 Rate of repeat cardiovascular events | **5** | **5** |  |
| -2-3 The rate of rehospitalization | **5** | **5** |  |
| -2-4 Mortality rate | **5** | **5** |  |
| -3 Health-care burden status |  |  |  |
| -3-1 length of hospital stay | **5** | **5** |  |
| -3-2 for hospitalization costs | **5** | **5** |  |
| Adventitia-4 satisfaction |  |  |  |
| -4-1 Patient satisfaction | **5** | **5** |  |
| -4-2 Physician satisfaction | **5** | **5** |  |
